# Psychobiological Correlates of Perceived Physical Activity Barriers: Insomnia, Chronotype, and Caffeine Consumption

**DOI:** 10.3390/ijerph23050666

**Published:** 2026-05-19

**Authors:** Mehmet Emre Eryücel, Mustafa Akil

**Affiliations:** Faculty of Sport Sciences, Akdeniz University, Antalya 07070, Türkiye; emreeryucel@akdeniz.edu.tr

**Keywords:** physical activity barriers, insomnia, chronotype, caffeine consumption

## Abstract

**Highlights:**

**Public health relevance—How does this work relate to a public health issue?**

- Physical inactivity and perceived participation barriers are common among university students.
- Sleep disturbances, chronotype, and caffeine use may be related to activity-related perceptions and daily functioning in young adults

**Public health significance—Why is this work of significance to public health?**

- The findings indicate that sleep-related and chronobiological factors show only weak and domain-specific associations with perceived physical activity barriers.

**Public health implications—What are the key implications or messages for practitioners, policy makers and/or researchers in public health?**

- Physical activity barriers in university populations appear to reflect multifactorial influences beyond sleep-related and chronobiological characteristics.
- Future longitudinal and objective-monitoring studies are needed to clarify temporal and behavioural mechanisms underlying perceived activity barriers.

**Abstract:**

Physical activity participation in young adulthood is typically explained by motivational and environmental determinants; however, regulatory models of daily behaviour suggest that transient fluctuations in sleep quality, circadian preference, and stimulant use may also be associated with how individuals appraise effort-related demands. Within this behavioural–temporal regulatory perspective, perceived barriers to physical activity may be related to variations in functional energy, alertness, and temporal alignment rather than solely stable contextual constraints. The present cross-sectional study examined whether insomnia symptoms (sleep initiation and awakening problems), chronotype, and daily caffeine intake were concurrently related to perceived personal, social, and environmental physical activity barriers in 788 university students (18–27 years). Standardized self-report measures were administered under controlled assessment conditions. Pearson correlations and theory-informed hierarchical regression models were applied. Sleep initiation problems demonstrated very weak positive correlations with total and domain-specific barriers (r = 0.12–0.17), whereas awakening problems showed very weak inverse correlations (r = −0.10 to −0.14, *p* ≤ 0.005). Chronotype was weakly associated only with personal barriers (β ≈ −0.09, *p* = 0.013). Daily caffeine intake showed a weak negative association with environmental barriers (β ≈ −0.15, *p* < 0.001). Across models, explained variance remained limited (adjusted R^2^ = 0.040–0.053), indicating that these variables explained only a very small proportion of variance in perceived physical activity barriers. These findings suggest that sleep-related and chronobiological characteristics are not meaningful independent predictors of perceived physical activity barriers in this population and demonstrate only weak, domain-specific, and non-directionally consistent associations. Accordingly, the findings should be interpreted cautiously as exploratory rather than practically predictive. Given the cross-sectional design and low explained variance, the results primarily highlight the limited explanatory utility of these psychobiological factors relative to broader unmeasured contextual determinants. Longitudinal and time-sensitive designs incorporating objective behavioural assessments are required to clarify temporal ordering and potential regulatory mechanisms.

## 1. Introduction

Young adulthood represents a distinctive life stage in which engagement in physical activity becomes closely intertwined with daily biological rhythms and energy regulation [1]. Global data indicate a rising trend in physical inactivity among individuals aged 18–29 years, with a substantial proportion of university students failing to meet recommended activity levels [2]. The transition to university life is characterised by increased academic demands, irregular daily schedules, and greater sedentary time, conditions that may intensify perceptions of constraint surrounding physical activity [3]. During this period, appraisals of activity-related demands may be shaped less by stable preferences and more by fluctuations in daily energy levels and temporal pressures [4]. Disruptions in sleep–wake patterns and daytime fatigue profiles may therefore be associated with how individuals perceive the feasibility, effort, and accessibility of engaging in physical activity [5]. The close linkage between perceived time availability, daily functioning, and evaluations of activity-related constraints underscores the importance of examining perceived physical activity barriers within this population [6,7].

Physical activity barriers have traditionally been conceptualised as relatively stable structural and motivational constraints, including limited time, environmental limitations, and insufficient motivational resources [8,9]. Reviews focusing specifically on university students indicate that perceived constraints surrounding physical activity are most often framed within these structural and individual domains [3]. Individual barriers commonly include reduced motivation, low perceived energy, poor self-regulatory capacity, and perceived lack of time for physical activity participation [3,9]. Social barriers are frequently associated with limited peer support, insufficient family encouragement, and reduced social reinforcement for participation in physical activity [8]. Environmental barriers typically involve restricted access to facilities, transportation difficulties, safety concerns, and limited opportunities for structured activity engagement [3,8]. These domains may also be associated with fluctuations in daily functioning and behavioural regulation, thereby corresponding to differences in how individuals appraise activity-related demands and accessibility [9,10]. However, more recent evidence suggests that barrier perceptions may also reflect conditions that fluctuate in daily life, particularly those related to energy regulation, sleep patterns, and behavioural timing [10]. The high prevalence of insomnia symptoms among university students is associated with reduced daytime functioning and diminished perceived energy availability [11], factors that may be related to how activity-related demands are appraised. Similarly, fatigue patterns and irregular sleep habits may be associated with perceptions of effort, feasibility, and time adequacy in relation to physical activity [12]. Among individuals who are new to physical activity or who lead sedentary lifestyles, sensations such as exercise-induced discomfort may be cognitively interpreted as barriers to activity [13]. Daily stimulant consumption, particularly caffeine and energy drinks, may further interact with these processes through its associations with sleep timing and perceived alertness [14]. The co-occurrence of these fluctuating behavioural and physiological states therefore suggests that perceived physical activity barriers may be embedded within dynamic daily regulatory processes rather than being solely determined by stable contextual factors [15].

Within this regulatory perspective, insomnia in young adulthood may be understood not only as a health indicator but also as a functional condition related to daily energy management and behavioural capacity [16]. Insufficient or poor-quality sleep is associated with reduced perceived energy levels during the day, which may be related to physiological and cognitive readiness for physical activity [17]. In addition, insomnia-related alterations in executive functioning may be associated with differences in planning, sustained attention, and goal-directed behaviour [18]. Such alterations in daily self-regulatory capacity may be associated with how individuals perceive and respond to physical activity demands [19]. Studies conducted among university students further indicate that insomnia is associated with disruptions in daily functioning and behavioural consistency [20]. From this perspective, insomnia may be viewed less as a purely clinical condition and more as a functional state associated with the temporal organisation of everyday behaviour [21]. This temporal dimension provides an important context for understanding how perceived physical activity barriers may be related to biological and behavioural rhythms [22].

Chronotype represents a relatively stable individual difference in circadian phase preference, reflecting variations in the timing of sleep–wake regulation and daily physiological alertness [23]. Research has predominantly examined chronotype in relation to overall physical activity levels or sedentary behaviour [24]. More recent work suggests that chronotype may also be associated with the timing and accessibility of behavioural opportunities, including physical activity engagement [25]. For example, individuals with an evening preference may experience a temporal misalignment between peak alertness and socially structured activity schedules, which may be reflected in perceived difficulty engaging in morning physical activity [26]. Evidence from university populations suggests that such timing-related mismatches may be linked to activity patterns and behavioural regularity [27]. In this sense, chronotype may be understood as a contextual characteristic associated with behavioural timing rather than as a direct determinant of physical activity behaviour [28]. This interpretation highlights the relevance of circadian organisation when examining perceived constraints on activity participation [29].

Caffeine consumption has most often been examined in relation to its acute effects on alertness, performance, and perceived fatigue [30]. Experimental and observational research indicates that caffeine temporarily increases alertness through adenosine antagonism [31]. At the same time, regular caffeine intake has been associated with delayed sleep initiation problems and alterations in subjective sleep quality, particularly when consumed later in the day [32]. Studies among university students have reported associations between caffeine intake and shorter sleep duration or reduced sleep efficiency [14]. Because caffeine may transiently attenuate perceived fatigue, it may also be associated with how individuals appraise their daily functional capacity, effort tolerance, or perceived time availability [33,34]. Accordingly, caffeine intake may be conceptualised not only as a performance-related substance but also as a short-term regulatory factor operating at the interface of sleep, alertness, and effort perception, and thus as a theoretically relevant correlate when examining fluctuations in perceived physical activity barriers [35].

Although sleep disturbances, chronotype, and stimulant use have each been linked to aspects of daily functioning, they are typically examined separately within physical activity research. Contemporary models of behavioural regulation suggest that evaluations of effort, feasibility, and time adequacy may be associated with dynamic interactions between physiological state, temporal alignment, and perceived functional capacity. From this perspective, perceived physical activity barriers may be related not only to stable structural or motivational constraints but also to context-dependent fluctuations in functional capacity associated with sleep quality, circadian phase preference, and state-dependent alertness. Insomnia-related disturbances may be associated with perceived energy availability and self-regulatory capacity; chronotype may be related to alignment between biological rhythms and socially structured schedules; and caffeine intake may be associated with alertness and fatigue appraisal. Rather than functioning independently, these processes may show concurrent associations with how activity-related demands are cognitively appraised under changing physiological and temporal conditions. The present study therefore examined whether insomnia symptoms (sleep initiation problems and awakening problems), chronotype, and daily caffeine intake are concurrently associated with perceived personal, social, and environmental barriers to physical activity among university students. Without assuming directional or causal relationships, the study tested whether these behavioural and chronobiological factors show domain-specific associations within a single analytical model. It was hypothesised that sleep initiation problems would be positively associated with perceived barriers, that chronotype would demonstrate context-dependent associations, and that caffeine intake would show limited and domain-specific relationships.

## 2. Materials and Methods

### 2.1. Ethical Approval and Study Design

This study involving human participants was conducted in accordance with fundamental ethical principles and the current guidelines of the Declaration of Helsinki. All participants were provided with detailed information regarding the purpose, scope, and procedures of the study. Written informed consent was obtained from all individuals who agreed to participate. The study protocol was reviewed and approved by the Ethics Committee (Approval No. 2026-22).

### 2.2. Sample, Participants, and Selection Procedure

**Figure 1:**
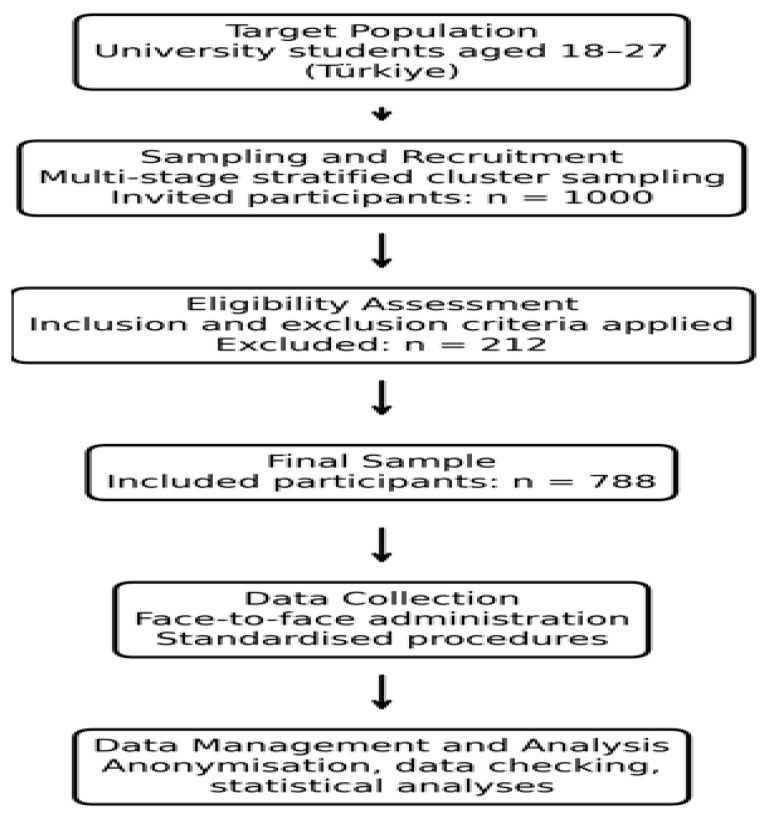
Flow diagram of participant recruitment, data collection, and analysis.

The target population of the study consisted of university students aged 18–27 years enrolled at public and foundation universities located in different regions of Türkiye. A multi-stage stratified cluster sampling framework based on geographical regions and university type (public or foundation) was used during participant recruitment. In the first stage, stratification was performed according to geographical regions and university type. In the second stage, universities were randomly selected from each stratum. In the third stage, participants were invited through faculty-, department-, or class-based clusters within the selected universities. Accordingly, the sampling process was conducted within a probabilistic framework; however, participation was voluntary and the final sample reflected a convenience-based participant structure. A total of 1000 students were invited to participate in the study, and 788 students completed all assessments without any missing data (response rate: 78.8%). No formal non-response bias analysis was performed. In line with the predefined exclusion criteria, 212 students were excluded from the study. The inclusion criteria were defined as having no history of chronic disease, no significant weight loss within the previous six months, and providing written informed consent to participate. Exclusion criteria included the presence of a diagnosed psychiatric disorder, being pregnant or breastfeeding, and regular use of medications known to affect metabolism or appetite. All inclusion and exclusion criteria were specified in detail in the study protocol prior to data collection and were systematically verified for each participant using a standardised checklist during the implementation phase (Figure 1).

### 2.3. Data Collection, Assessment Conditions, and Data Security

All measurements were conducted in accordance with standardised procedures and under controlled environmental conditions. The data collection process was carried out within the same day to ensure that participants were assessed within comparable time frames. Participants were instructed to refrain from vigorous exercise, alcohol consumption, and high caffeine intake during the 24 h preceding the assessments. All procedures were performed by the same research team, and the assessors received training on shared protocols prior to data collection. In cases where multiple assessors were involved, inter-rater consistency was monitored at regular intervals. Participants were provided with neutral and standardised instructions, the clarity of which had been tested in advance through a pilot study. Collected data were recorded using anonymous identification codes to protect participant confidentiality. Data entry was verified using a double-check method, and any potential errors or missing values were identified and corrected on the same day. These procedures were implemented to minimise measurement errors and selection bias and to enhance the validity and reliability of the study findings.

### 2.4. Data Collection Flow

In this study, the data collection process was conducted face to face, and all participants were assessed during a single session following a standardised sequence. Data collection was performed separately within each participating faculty under the supervision of the same research team, and participants from the same assessment setting were evaluated simultaneously in order to minimise communication and interaction between groups during the assessment process. First, demographic and lifestyle information, including age and sex, was collected. Subsequently, the Insomnia Complaints and Basic Sleep Quality Scale (ICBSQS) was administered to assess sleep-related characteristics. This was followed by the Morningness–Eveningness Questionnaire (MEQ) to determine chronotype, the Physical Activity Participation Barriers Scale (PAPBS) to evaluate individual, social, and environmental factors limiting engagement in physical activity, and the Caffeine Consumption Frequency Record Form to assess levels of caffeine intake. All assessments were carried out under controlled environmental conditions and in accordance with standardised instructions, thereby minimising inter-measurement variability and assessor-related error. The internal consistency of the data obtained from the scales was evaluated using Cronbach’s alpha coefficients, and the distributional properties of the variables were deemed appropriate for parametric analyses.

### 2.5. Instruments and Measures

#### 2.5.1. Insomnia Complaints and Basic Sleep Quality Scale

The Insomnia Complaints and Basic Sleep Quality Scale was developed by Allen Gomes et al. (2015) to assess sleep disturbances and sleep quality [36]. It is a brief and easily administered self-report instrument consisting of seven items. The scale covers core components of sleep, including sleep onset latency and difficulty initiating sleep, frequency of nocturnal awakenings, early morning awakening, whether awakenings during the night or early morning are perceived as problematic, as well as subjective sleep quality and sleep depth. The Turkish validity and reliability study of the scale was conducted by Mıhçıoğlu et al. (2021) [37], who reported an internal consistency coefficient of Cronbach’s alpha = 0.752. In the present study, exploratory factor analysis (EFA) was conducted to examine the structural stability of the scale within the current sample and to verify whether the previously reported factor configuration was reflected in this population. The results of the Kaiser–Meyer–Olkin measure and Bartlett’s test of sphericity indicated that the data were suitable for factor analysis. The analyses supported the retention of the expected two-factor configuration (Sleep Initiation Problems and Awakening Problems), with item factor loadings ranging from 0.50 to 0.80 on their respective factors. Internal consistency of the scale was re-evaluated in the present sample, yielding a Cronbach’s alpha coefficient of 0.814. In addition, the distributional properties of the total and subscale scores were within acceptable ranges, and the assumptions required for parametric analyses were considered to be satisfied.

#### 2.5.2. Physical Activity Participation Barriers Scale

In this study, the Physical Activity Participation Barriers Scale (PAPBS) was used to provide a comprehensive assessment of individual, social, and environmental factors that limit engagement in physical activity. The original version of the scale was developed by Ibrahim et al. (2013) and consists of 24 items across three sub-dimensions: personal barriers, social environment, and physical environment [38]. The personal barriers dimension includes factors such as low motivation, fatigue, fear of injury, lack of self-discipline, and negative perceptions regarding exercise participation. The social environment dimension reflects limited encouragement from family or friends, lack of exercise partners, and perceived social support deficiencies. The physical environment dimension includes barriers related to limited access to facilities, transportation difficulties, environmental safety concerns, weather conditions, and financial limitations associated with physical activity participation [38,39]. Representative items include statements such as “I do not have enough energy to engage in physical activity after work,” “My family/friends do not encourage me to be physically active,” and “There are no suitable facilities or spaces for physical activity in my living environment” [39]. The Turkish adaptation of the scale was conducted by Yurtçiçek et al. (2018) [39]. In the adaptation study, the scale was reported to be suitable for factor analysis (KMO = 0.84; Bartlett’s χ^2^(231) = 2346.96) and to demonstrate adequate internal consistency (α = 0.87). In the present study, exploratory factor analysis (EFA) was conducted to examine the structural consistency of the scale within the current sample and to evaluate whether the previously reported factor configuration was reflected in this population. The results supported the retention of the expected three-factor structure, with item factor loadings ranging from 0.41 to 0.76 across the respective factors and a total explained variance of 58%. Internal consistency was recalculated in the present sample, yielding a Cronbach’s alpha coefficient of 0.91 for the total scale. Reliability coefficients for the sub-dimensions were 0.88 for personal barriers, 0.79 for social environment, and 0.84 for physical environment. The distributional properties of the total and subscale scores were also examined. Skewness and kurtosis values fell within acceptable ranges, indicating that the assumptions required for parametric analyses were met. Overall, these findings indicate that the scale demonstrated satisfactory structural consistency and internal reliability in the current sample, supporting its suitability for use in the analytical models of the study.

#### 2.5.3. Caffeine Consumption Frequency Record Form

In this study, the Caffeine Consumption Frequency Record Form was used to estimate participants’ daily caffeine intake. The form, originally described by Otman (2017) [40], requires individuals to report the frequency and typical portion size of tea, coffee, energy drinks, cola, chocolate, and other caffeine-containing products consumed over the previous month. Reported intake was converted into milligrams using the Turkish Nutrition Guidelines (TÜBER) and internationally recognised caffeine content reference tables, and average daily caffeine intake (mg/day) was calculated. Although the assessment is based on self-report, this structured frequency approach enables standardised quantification of intake across different beverage types and portion sizes, facilitating comparability between participants. Similar questionnaire-based methods have demonstrated acceptable validity and reliability in adolescent and university populations [41]. A one-month reporting window was selected to capture typical consumption patterns rather than short-term fluctuations. For descriptive purposes, daily caffeine intake was additionally classified into three categories based on commonly used cut-off values: low (≤150 mg/day), moderate (150–300 mg/day), and high (>300 mg/day). In the primary regression analyses, caffeine intake was modelled as a continuous variable (mg/day) to preserve statistical power and avoid information loss.

#### 2.5.4. Morningness–Eveningness Questionnaire (MEQ)

In this study, the Morningness–Eveningness Questionnaire (MEQ) was used to determine participants’ chronotype characteristics and to examine the relationship between these characteristics and physical activity participation. The MEQ is grounded in circadian rhythm theory and assesses individuals’ diurnal patterns of physiological alertness, performance tendencies, and activity preferences along the morningness–eveningness continuum. Originally developed by Horne and Östberg (1976) [42], the MEQ is a unidimensional self-report instrument consisting of 19 items, with total scores ranging from 16 to 86, allowing for the classification of individuals’ chronotype. Based on established scoring criteria, participants were classified as definitely evening type (16–30), moderately evening type (31–41), intermediate type (42–58), moderately morning type (59–69), or definitely morning type (70–86). The Turkish adaptation of the questionnaire was conducted by Pündük et al. (2005) [43]. In the adaptation study, reported Cronbach’s alpha coefficients (0.78–0.81) and test–retest reliability (r = 0.84) demonstrated adequate internal consistency and temporal stability. Owing to its unidimensional structure, factor analysis was not required. The MEQ therefore enabled the inclusion of chronotype as a biological variable in the statistical models of the study. In this respect, the instrument is directly aligned with the theoretical framework underpinning the examination of chronotype-related behavioural patterns associated with physical activity [42,43].

### 2.6. Statistical Analysis

All statistical analyses were conducted using IBM SPSS Statistics (Version 26.0; IBM Corp., Armonk, NY, USA). The dataset was screened for missing values, outliers, and distributional characteristics prior to analysis. Standardised residuals and residual plots were additionally examined to evaluate the potential influence of outliers on the regression models, and no cases demonstrating disproportionate influence were identified. Descriptive statistics are presented as means, standard deviations, frequencies, and percentages. Exploratory factor analysis (EFA) was performed to examine whether the previously reported factor structures of the Insomnia Complaints and Basic Sleep Quality Scale and the Physical Activity Participation Barriers Scale were replicated in the present sample. Sampling adequacy was assessed using the Kaiser–Meyer–Olkin (KMO) measure and Bartlett’s test of sphericity. A factor loading threshold of 0.40 was applied. The expected two-factor structure for insomnia symptoms and the three-factor structure for physical activity barriers were retained. Internal consistency was evaluated using Cronbach’s alpha coefficients (α = 0.81–0.92). Skewness and kurtosis values were within ±1.5. Given the large sample size (*n* = 788), parametric tests were applied. Pearson’s product–moment correlation coefficients were calculated to examine bivariate relationships. Hierarchical multiple linear regression analyses were conducted to evaluate associations of chronotype, sleep initiation problems, awakening problems, and caffeine intake with the sub-dimensions of physical activity barriers. Chronotype, insomnia subcomponents, and caffeine intake were entered sequentially into the models to examine their relative statistical contributions. Multicollinearity was assessed using variance inflation factor (VIF) values, which ranged from 1.00 to 1.47. Daily caffeine intake was analysed as a continuous variable (mg/day) in the regression models. Statistical significance was set at *p* < 0.05 (two-tailed). Given the number of statistical comparisons and the relatively small effect sizes observed, statistically significant findings were interpreted cautiously.

## 3. Results

### 3.1. Participant Characteristics

A total of 788 participants were included in the study, of whom 49.4% were female (*n* = 389) and 50.6% were male (*n* = 399). The age distribution was as follows: ≤20 years: 56.9% (*n* = 448); 21–23 years: 29.3% (*n* = 231); and 24–27 years: 13.8% (*n* = 109). Chronotype distribution was 7.1% definitely morning type (*n* = 56), 13.2% morning type (*n* = 104), 50.9% intermediate type (*n* = 401), 16.6% evening type (*n* = 131), and 12.2% definitely evening type (*n* = 96). The mean age of the participants was 20.89 ± 2.92 years, with a mean body mass of 70.44 ± 17.13 kg and a mean height of 170.89 ± 9.65 cm. Daily caffeine consumption was classified as low (48.48 ± 24.64 mg/day; *n* = 213), moderate (148.19 ± 29.20 mg/day; *n* = 192), moderate–high (247.31 ± 26.71 mg/day; *n* = 182), and high (345.16 ± 28.57 mg/day; *n* = 201). The total insomnia symptom score was 1.68 ± 0.43, with mean scores of 1.85 ± 0.69 for sleep initiation problems and 1.51 ± 0.53 for awakening problems. The total physical activity barriers score was 2.08 ± 0.73, while mean scores for the sub-dimensions were 2.07 ± 0.75 for personal barriers, 2.03 ± 0.91 for social barriers, and 2.14 ± 0.87 for environmental barriers. Overall, the sample comprised a predominantly young university student population with a balanced sex distribution and a chronotype profile largely characterised by intermediate-type tendencies (Table 1).

### 3.2. Correlation Analysis

Pearson correlation analyses (Table 2) indicated small positive associations between sleep initiation problems and physical activity barriers across all domains (r = 0.119–0.165, *p* ≤ 0.001). In contrast, awakening problems were weakly and inversely associated with total and sub-dimension barrier scores (r = −0.101 to −0.140, *p* ≤ 0.005). The total insomnia score was not significantly associated with physical activity barriers (*p* > 0.05). Chronotype only showed a small negative association with personal barriers (r = −0.089, *p* = 0.013). Caffeine intake demonstrated a weak negative association with environmental barriers (r = −0.127, *p* < 0.001), while no significant associations were observed with other barrier dimensions or the total barrier score. Overall, all correlation coefficients were small in magnitude, indicating limited bivariate associations between sleep-related variables, chronotype, caffeine intake, and perceived physical activity barriers (Table 2).

### 3.3. Hierarchical Regression Analysis

Hierarchical regression analyses (Table 3) examined the independent contributions of chronotype, insomnia subcomponents, and caffeine intake to physical activity barrier dimensions. For personal barriers, chronotype showed a small negative association in Model 1 (β = −0.089, *p* = 0.013). After inclusion of insomnia variables, sleep initiation problems were positively associated (β = 0.174, *p* < 0.001) and awakening problems negatively associated (β = −0.137, *p* < 0.001). Caffeine intake was not significant. For social barriers, chronotype was not significant in Model 1 but became significant after the inclusion of insomnia variables (β = 0.135, *p* = 0.002). Sleep initiation problems (β = 0.191, *p* < 0.001) and awakening problems (β = −0.151, *p* < 0.001) were consistently associated, whereas caffeine intake was not significant. For environmental barriers, sleep initiation problems (β = 0.197, *p* < 0.001) and awakening problems (β = −0.105, *p* = 0.004) were significant predictors. In the final model, caffeine intake showed an additional small negative association (β = −0.151, *p* < 0.001), while chronotype was not significant. Across models, explained variance ranged from 4% to 5% (adjusted R^2^ = 0.040–0.053). VIF values (1.00–1.47) indicated no multicollinearity concerns (Table 3).

## 4. Discussion

Sleep initiation problems showed the most consistent very weak associations across barrier domains, suggesting that difficulties in initiating sleep may be associated with increased perceptions of effort, time constraints, or environmental obstacles related to physical activity. In contrast, awakening problems demonstrated inverse associations with barriers, indicating that different components of insomnia symptoms may reflect heterogeneous patterns rather than a uniform sleep-related burden. Notably, the total insomnia score was not significantly associated with barrier dimensions, highlighting the importance of examining symptom subcomponents rather than aggregated sleep indices. Chronotype demonstrated limited direct associations in bivariate analyses, and its significance emerged primarily after adjustment for insomnia-related symptoms, suggesting possible shared variance or statistical suppression effects between sleep timing and perceived barriers. Caffeine intake showed a small and domain-specific association with environmental barriers only, although this finding should be interpreted cautiously given the low explained variance observed across models.

The consistent association between sleep initiation problems and higher perceived barriers across personal, social, and environmental domains may reflect the broader functional correlates of difficulties initiating sleep [16]. Sleep initiation problems have been linked to alterations in executive functioning, attentional continuity, and planning processes [18], which may be associated with differences in how individuals appraise effort and organise goal-directed behaviours such as physical activity [44]. In this context, variations in perceived energy and regulatory capacity may be related to evaluations of activity-related demands. However, given the small effect sizes observed, sleep initiation problems should be interpreted as one contributory factor rather than a primary determinant of perceived barriers. The inverse associations between awakening problems and barrier dimensions require cautious interpretation [20]. This pattern may reflect heterogeneity in sleep experiences, measurement-related characteristics of the awakening subscale, shared variance between variables, or chance findings [17,45]. Because the study employed a cross-sectional design, temporal ordering cannot be determined, and reciprocal or bidirectional relationships between sleep characteristics and activity-related perceptions remain possible.

The association between chronotype and physical activity barriers was limited and domain-specific. The small negative correlation with personal barriers (r = −0.089) indicates that chronotype only accounted for a minimal proportion of variance at the bivariate level [24]. In regression analyses, chronotype reached statistical significance only after adjustment for insomnia-related symptoms, suggesting that its association may reflect shared variance with sleep characteristics or possible statistical suppression effects rather than a clear independent relationship [23]. These findings align with conceptualisations of chronotype as a relatively stable circadian phase preference associated with temporal alignment between biological rhythms and socially structured schedules, rather than directly determining behavioural engagement [46,47]. Within university environments characterised by externally regulated time demands, the potential association between chronotype and perceived activity barriers may therefore be attenuated by situational constraints and overlapping sleep-related factors [27]. Accordingly, chronotype should be interpreted as a variable showing limited and context-dependent associations within the present models rather than as a primary explanatory factor [29].

In the present study, caffeine intake demonstrated a small negative association with physical environmental barriers (β = −0.151), indicating a limited and domain-specific relationship with perceived activity constraints [14]. This finding suggests that caffeine consumption may be associated with differences in the appraisal of environmental demands rather than reflecting a broad reduction in perceived barriers. Experimental evidence indicates that caffeine can temporarily enhance alertness [31], which may be related to short-term evaluations of effort or environmental demands without necessarily reflecting sustained behavioural patterns [33]. Given the modest effect size and the low proportion of explained variance across models, caffeine intake should be interpreted as a contextual correlate rather than an independent explanatory factor in perceived physical activity barriers. In addition, the domain-specific nature of this finding should be interpreted cautiously, as it may reflect measurement-related variability or chance associations within the present dataset.

This study has several limitations. First, the cross-sectional design does not permit causal inference; the findings reflect concurrent associations only, and reciprocal or reverse relationships between sleep characteristics, caffeine intake, and perceived physical activity barriers remain plausible. Second, all variables were assessed using self-report measures, which may introduce recall bias, reporting inaccuracies, and social desirability effects. In addition, the timing of caffeine intake was not recorded. Given that caffeine effects are time-dependent, this limits interpretation of its associations with sleep regulation and perceived daily functioning. Morning versus evening caffeine consumption may show differential associations with sleep quality, alertness, and perceived activity-related demands; therefore, analysing caffeine intake solely as total daily consumption (mg/day) may have obscured potentially meaningful timing-related patterns. Third, the relatively low proportion of explained variance indicates that physical activity barriers are influenced by additional unmeasured factors. These may include academic workload, socioeconomic context, environmental infrastructure, time management capacity, objectively measured physical activity levels, and chronobiological indicators such as social jetlag or day-to-day variability in sleep timing. In addition, the sample consisted exclusively of university students from Türkiye, which may limit the generalisability of the findings to other cultural, educational, or institutional contexts. Perceptions of physical activity barriers, chronotype-related behaviours, and caffeine consumption patterns may vary across sociocultural settings and university systems. No formal correction for multiple statistical comparisons (e.g., Bonferroni adjustment) was applied in the present analyses. Consequently, some statistically significant findings, particularly those with marginal *p*-values, may reflect type I error and should therefore be interpreted cautiously. Given the relatively small effect sizes and low explained variance observed across models, the findings are better interpreted as exploratory and hypothesis-generating rather than confirmatory evidence of robust explanatory relationships. Despite these limitations, the study has notable strengths. The large sample size and balanced chronotype distribution enhance the robustness of the findings. The simultaneous examination of insomnia subcomponents, chronotype, and caffeine intake within a single analytical model allows for a more differentiated assessment of their joint associations. Moreover, systematic evaluation of model assumptions supports the stability of the reported estimates. Overall, the findings should be interpreted as hypothesis-generating rather than as direct grounds for intervention. The consistent association between sleep initiation problems and perceived barriers highlights the need for longitudinal and experimental research to determine whether sleep-related processes are prospectively associated with activity-related perceptions. Future studies incorporating objective physical activity measures, timing-sensitive caffeine assessments, and broader chronobiological indicators may further clarify these relationships.

## 5. Conclusions

This study indicates that sleep initiation and awakening problems show very weak and concurrent associations with perceived physical activity barriers among university students, whereas chronotype demonstrates limited and context-dependent relationships and caffeine intake is only associated with environmental barriers. The small effect sizes and low explained variance suggest that these variables only account for a limited proportion of the multifactorial processes underlying perceived activity constraints. Accordingly, the findings should be interpreted as indicative of concurrent associations rather than dominant explanatory mechanisms. Given the cross-sectional design and limited predictive utility of the models, the observed domain-specific patterns should be interpreted cautiously and may reflect shared variance, measurement-related variability, or chance findings. Future research incorporating objective physical activity measures and longitudinal or experimental designs is needed to clarify temporal ordering and to better understand how sleep- and timing-related factors interact with broader contextual determinants of physical activity barriers.

## Figures and Tables

**Table 1:** Descriptive Characteristics, Frequencies, and Study Variables.

Variable	Category	N	Mean	SD/%
Gender	Female	389		49.4%
	Male	399		50.6%
Age Group	≤20	448		56.9%
	21–23	231		29.3%
	≥24–27	109		13.8%
Chronotype	Definitely morning	56		7.1%
	Morning type	104		13.2%
	Intermediate	401		50.9%
	Evening type	131		16.6%
	Definitely evening	96		12.2%
Age (years)		788	20.89	2.92
Body weight (kg)		788	70.44	17.13
Height (cm)		788	170.89	9.65
Caffeine intake (mg/day)	Low caffeine consumption	213	48.48	24.64
	Moderate caffeine consumption	192	148.19	29.20
	Moderate-to-high caffeine consumption	182	247.31	26.71
	High caffeine consumption	201	345.16	28.57
Insomnia symptoms (total)		788	1.68	0.43
Sleep initiation problems		788	1.85	0.69
Awakening problems		788	1.51	0.53
Physical activity barriers (total)		788	2.08	0.73
Personal barriers		788	2.07	0.75
Social barriers		788	2.03	0.91
Environmental barriers		788	2.14	0.87

**Table 2:** Correlation of Sleep, Chronotype, and Caffeine With Physical Activity Barriers.

Variable	Statistic	PA Barriers (Total)	Personal Barriers	Social Barriers	Environmental Barriers
Insomnia (Total)	r	0.045	0.043	0.016	0.059
	95% CI	[−0.02, 0.11]	[−0.03, 0.11]	[−0.05, 0.09]	[−0.01, 0.13]
	*p*-value	0.208	0.227	0.651	0.100
Sleep initiation problems	r	0.165 **	0.160 **	0.119	0.151 **
	95% CI	[0.1, 0.23]	[0.09, 0.23]	[0.05, 0.19]	[0.08, 0.22]
	*p*-value	<0.001	<0.001	0.001	<0.001
Awakening problems	r	−0.140 **	−0.137 **	−0.127 **	−0.101
	95% CI	[−0.21, −0.07]	[−0.2, −0.07]	[−0.2, −0.06]	[−0.17, −0.03]
	*p*-value	<0.001	<0.001	<0.001	0.005
Chronotype	r	−0.046	−0.089	0.008	−0.047
	95% CI	[−0.12, 0.02]	[−0.16, −0.02]	[−0.06, 0.08]	[−0.12, 0.02]
	*p*-value	0.197	0.013	0.816	0.187
Caffeine intake	r	−0.067	−0.025	−0.022	−0.127 **
	95% CI	[−0.14, 0.0]	[−0.09, 0.04]	[−0.09, 0.05]	[−0.2, −0.06]
	*p*-value	0.058	0.491	0.544	<0.001

Pearson correlation coefficients are reported with 95% confidence intervals. ** *p* < 0.01 (two tailed).

**Table 3:** Predictors of Physical Activity Barriers: Hierarchical Regression Analysis Results.

Dependent Variable	M	Predictor	B	SE	β	t	*p*	95% CI	VIF
Personal Barriers	1	Chronotype	−0.065	0.026	−0.089	−2.498	0.013	[−0.116, −0.014]	1.000
		Model 1 Summary: R = 0.089, R^2^ = 0.008, Adj. R^2^ = 0.007, Cohen’s f^2^ = 0.008							
	2	Sleep initiation problems	0.187	0.045	0.172	4.168	<0.001	[0.099, 0.275]	1.391
	2	Awakening problems	−0.195	0.051	−0.138	−3.816	<0.001	[−0.295, −0.095]	1.070
		Model 2 Summary: R = 0.208, R^2^ = 0.043, Adj. R^2^ = 0.040, Cohen’s f^2^ = 0.045							
	3	Sleep initiation problems	0.190	0.045	0.174	4.221	<0.001	[0.102, 0.278]	1.393
	3	Awakening problems	−0.193	0.051	−0.137	−3.775	<0.001	[−0.293, −0.093]	1.070
	3	Caffeine intake	−0.018	0.017	−0.036	−1.028	0.304	[−0.051, 0.015]	1.002
		Model 3 Summary: R = 0.211, R^2^ = 0.045, Adj. R^2^ = 0.040, Cohen’s f^2^ = 0.047							
Social Barriers	1	Chronotype	0.007	0.031	0.008	0.233	0.816	[−0.054, 0.068]	1.000
		Model 1 Summary: R = 0.008, R^2^ = 0.000, Adj. R^2^ = −0.001, Cohen’s f^2^ = 0.001							
	2	Chronotype	0.126	0.037	0.143	3.371	0.001	[0.054, 0.198]	1.472
	2	Sleep initiation problems	0.246	0.054	0.187	4.549	<0.001	[0.140, 0.352]	1.391
	2	Awakening problems	−0.261	0.062	−0.153	−4.236	<0.001	[−0.383, −0.139]	1.070
		Model 2 Summary: R = 0.208, R^2^ = 0.043, Adj. R^2^ = 0.039, Cohen’s f^2^ = 0.045							
	3	Chronotype	0.119	0.038	0.135	3.177	0.002	[0.045, 0.193]	1.472
	3	Sleep initiation problems	0.252	0.054	0.191	4.644	<0.001	[0.146, 0.358]	1.393
	3	Awakening problems	−0.257	0.061	−0.151	−4.177	<0.001	[−0.377, −0.137]	1.070
	3	Caffeine intake	−0.034	0.021	−0.058	−1.650	0.099	[−0.075, 0.007]	1.002
		Model 3 Summary: R = 0.215, R^2^ = 0.046, Adj. R^2^ = 0.042, Cohen’s f^2^ = 0.048							
Physical Environmental Barriers	1	Chronotype	−0.040	0.030	−0.047	−1.320	0.187	[−0.099, 0.019]	1.000
		Model 1 Summary: R = 0.047, R^2^ = 0.002, Adj. R^2^ = 0.001, Cohen’s f^2^ = 0.002							
	2	Chronotype	0.065	0.036	0.077	1.816	0.070	[−0.005, 0.135]	1.472
	2	Sleep initiation problems	0.236	0.052	0.187	4.521	<0.001	[0.134, 0.338]	1.391
	2	Awakening problems	−0.181	0.059	−0.111	−3.053	0.002	[−0.297, −0.065]	1.070
		Model 2 Summary: R = 0.189, R^2^ = 0.036, Adj. R^2^ = 0.032, Cohen’s f^2^ = 0.037							
	3	Chronotype	0.049	0.036	0.058	1.361	0.174	[−0.022, 0.120]	1.472
	3	Sleep initiation problems	0.249	0.052	0.197	4.815	<0.001	[0.147, 0.351]	1.393
	3	Awakening problems	−0.172	0.059	−0.105	−2.927	0.004	[−0.288, −0.056]	1.070
	3	Caffeine intake	−0.084	0.020	−0.151	−4.289	<0.001	[−0.123, −0.045]	1.002
		Model 3 Summary: R = 0.241, R^2^ = 0.058, Adj. R^2^ = 0.053, Cohen’s f^2^ = 0.062							

Note: B = unstandardized coefficient; SE = standard error; β = standardized coefficient; CI = 95% confidence interval. R, R^2^, and adjusted R^2^ are reported in the table for each model step. All tolerance values were above 0.65, indicating no multicollinearity concerns. Cohen’s f^2^ values indicated small effect sizes across all models.

## Data Availability

The data presented in this study are available from the corresponding author on reasonable request. The data are not publicly available due to privacy and ethical restrictions.
